# Improving the stability of the TetR/Pip-OFF mycobacterial repressible promoter system

**DOI:** 10.1038/s41598-019-42319-2

**Published:** 2019-04-08

**Authors:** Francesca Boldrin, Saber Anoosheh, Agnese Serafini, Laura Cioetto Mazzabò, Giorgio Palù, Roberta Provvedi, Riccardo Manganelli

**Affiliations:** 1 Department of Molecular Medicine, University of Padova, Padova, Italy; 2 Department of Biology, University of Padova, Padova, Italy; 3 Present Address: Institute of Infectious Disease and Molecular Medicine, University of Cape Town UCT, Cape Town, South Africa; 4 Present Address: Mycobacterial Metabolism and Antibiotic Research Laboratory, The Francis Crick Institute, London, UK

## Abstract

Tightly regulated gene expression systems are powerful tools to study essential genes and characterize potential drug targets. In a past work we reported the construction of a very stringent and versatile repressible promoter system for *Mycobacterium tuberculosis* based on two different repressors (TetR/Pip-OFF system). This system, causing the repression of the target gene in response to anhydrotetracycline (ATc), has been successfully used in several laboratories to characterize essential genes in different mycobacterial species both *in vitro* and *in vivo*. One of the limits of this system was its instability, leading to the selection of mutants in which the expression of the target gene was no longer repressible. In this paper we demonstrated that the instability was mainly due either to the loss of the integrative plasmid carrying the genes encoding the two repressors, or to the selection of a frameshift mutation in the gene encoding the repressors Pip. To solve these problems, we (i) constructed a new integrative vector in which the gene encoding the integrase was deleted to increase its stability, and (ii) developed a new integrative vector carrying the gene encoding Pip to introduce a second copy of this gene in the chromosome. The use of these new tools was shown to reduce drastically the selection of escape mutants.

## Introduction

During the last few years, several tightly regulated gene expression systems have been developed and successfully used in *Mycobacterium tuberculosis* to study essential genes and characterize potential drug targets^1–3^. The TetR/Pip-OFF system represents one of the most versatile and stringent of such systems^4^. It is based on two repressors, whose structural genes are integrated in single copy into the chromosome, and the *Streptomyces pristinaespiralis* tunable promoter P_*ptr*_. To modulate the expression of the target gene, its promoter must be replaced with P_*ptr*_, then an integrative plasmid carrying the genes encoding the P_*ptr*_ repressor (Pip) and the tetracycline-responsive repressor TetR is integrated at the mycobacteriophage L5 *attB* site. When bacteria are grown without anhydrotetracycline (ATc) TetR represses *pip* expression and the gene of interest is expressed. However, when ATc is added to the culture medium, the repressor Pip is expressed causing P_*ptr*_ repression. This system has been successfully used in several mycobacterial species *in vitro* and *in vivo* during mice infection^5–12^.

The main disadvantage of this otherwise very successful system was the strength of P_*ptr*_, leading to overexpression of target genes physiologically expressed at low level. This problem was recently overcome by the construction of a series of P_*ptr*_ mutants with different strength to generate conditional mutants expressing the target gene close to its physiological level^13^. A second problem of the TetR/Pip-OFF system was its instability: when an essential gene was placed under its transcriptional control, a small number of escape mutants whose target gene was no longer repressible and therefore able to grow in the presence of ATc were selected. Since this instability might represent a problem in long *in vivo* experiments, in this manuscript we investigated the reasons of this instability and developed a strategy to reduce it by using two conditional mutants previously constructed in our laboratory (*Mycobacterium smegmatis ftsZ* and *M*. *tuberculosis sigA* mutants).

## Materials and Methods

### Strain and culture conditions

The following bacterial strains were used in this study: *Escherichia coli* Top10 (Invitrogen, Carlsbad, CA, USA), DH5α (laboratory stock*)*, *M*. *smegmatis* mc^2^155 (laboratory stock), and *M*. *tuberculosis* H37Rv (laboratory stock). *E*. *coli* strains were grown at 37 °C in Luria-Bertani (LB) broth or on LB agar plates. Mycobacterial strains were grown at 37 °C in Middlebrook 7H9 broth (Difco, Franklin Lakes, NJ, USA) in 150 ml roller bottles with slow rotation (3 rpm), in 10 ml screw-cap tubes without agitation, or Middlebrook 7H10 agar plates (Difco) supplemented with 0.2% glycerol and 0.05% Tween-80. For growth of *M*. *tuberculosis*, the medium was supplemented with 10% albumin-dextrose-sodium chloride complex (ADN)^14^. When needed, antibiotics (Sigma) were added to the media at the following concentrations: streptomycin (Sm) 20 µg ml^−1^, kanamycin (Km) 50 µg ml^−1^ (*E*. *coli*) or 20 µg ml^−1^ (*M*. *smegmatis* and *M*. *tuberculo*sis), hygromycin (Hyg) 150 µg ml^−1^ (*E*. *coli*) or 50 µg ml^−1^ (*M*. *smegmatis* and *M*. *tuberculosis*), anhydrotetracycline (ATc) 50 ng ml^−1^ (*M*. *smegmatis*) or 500 ng ml^−1^ (*M*. *tuberculosis*). Preparation of electrocompetent cells for electroporation and preparation of mycobacterial genomic DNA were performed as previously described^15^. *M*. *tuberculosis* strains were handled and cultivated in a Biosafety Level 3 Laboratory (BLS3).

### Plasmids construction

To construct an integrative plasmid carrying the TetR/Pip-OFF system, but not the integrase, the DNA fragment containing the *attP* sequence of pMC1s^16^, obtained cutting the plasmid with *BamHI*, was treated with Klenow polymerase and cloned in the *ScaI* site of a pSM128^17^ derivative in which the region carrying the *attB* site and the integrase gene was previously removed using *NheI* and *HindIII*. The resulting plasmid was named pANTO4 (Fig. S1a). The DNA fragment encoding the TetR/Pip-OFF system belonging to pFRA61^4^ was then cloned at the unique ApaI site of pANTO4 to generate pANTO5, conferring resistance to Sm (Table 1 and Fig. S1b). To generate a replicative plasmid carrying the gene encoding the mycobacteriophage L5 integrase, the integrase gene of pMV306^16^ was cloned into the replicative plasmid pMV261^18^ after digestion with *NheI/KpnI* to generate pANTO3, conferring resistance to Km (Table 1 and Fig. S1c). Finally, to generate an integrative plasmid able to introduce a second copy of the *pip* gene in the chromosome of our conditional mutants, this gene was PCR amplified from pFRA38^4^ and cloned at the *EcoRI* site of pTTP1B^19^ to generate pAGN33 (Table 1 and Fig. S1d). pTTP1B is an integrative plasmid based on a mycobacteriophage (Tweety) able to recognize an *attB* site different from that recognized by the mycobacteriophage L5^19^.

**Table 1:** Plasmids used in this study.

Plasmids	Description
pANTO3	Replicative plasmid derived from pMV261 and carrying the mycobacteriophage L5 integrase gene, Km^R^
pANTO5	Integrative plasmid derived from pSM128 w/o integrase, carrying the mycobacteriophage L5 *attP* site and TetR/Pip-OFF system, Sm^R^
pAGN33	Integrative plasmid derived from pTT1B and carrying *pip*, Km^R^

### Strain construction

pANTO5 and pANTO3 were co-electroporated into Ms96^4^ and TB167. Ms96 is a *M*. *smegmatis* strain in which the *ftsZ* gene is transcriptionally controlled by P_*ptr*_, while TB167 is a *M*. *tuberculosis* strain in which the *sigA* gene is transcriptionally controlled by P_*ptr*_. The resulting strains, selected on Sm and Km were grown for some generations without Km selection and then plated on 7H10 containing Sm to allow selection of strains with pANTO5 integrated into the chromosome, but missing pANTO3. Some colonies were streaked on plates with Km to confirm the loss of pANTO3 and selected for further studies. The resulting strains were named Ms165 and TB213 respectively (Tables 2 and 3).

**Table 2:** *M*. *smegmatis* strains used in this study.

*M*. *smegmatis*	Parental strain	Relevant genotype	Reference
Ms 96	mc^2^155	P_*ptr*_-*ftsZ;* Hyg^R^	Boldrin 2010
Ms98	Ms96	P_*ptr*_-*ftsZ*, TetR- PipOFF system, *int* (cis); Hyg^R^, Sm^R^	Boldrin 2010
Ms163	Ms96	P_*ptr*_-*ftsZ*, TetR- PipOFF system, *int* (trans), pANTO3::pANTO5 HygR^R^, Km^R^, Sm^R^	This work
Ms165	Ms163	P_*ptr*_-*ftsZ*, TetR- PipOFF system::pANTO5 HygR^R^, Sm^R^	This work
Ms199	Ms165	P_*ptr*_-*ftsZ*,::pANTO5,::pAGN33 *pip* merodiploid, Hyg^R^, Sm^R^, Km^R^	This work

**Table 3:** *M*. *tuberculosis* strains used in this study.

*M*. *tuberculosis*	Parental strain	Relevant genotype	References
TB167	H37Rv	P_*ptr*_-*sigA;* Hyg^R^	Unpublished data
TB259	TB167	P_*ptr*_-*sigA*, TetR- PipOFF system, *int* (cis); Hyg^R^, Sm^R^	Unpublished data
TB212	TB259	P_*ptr*_-*sigA*, TetR- PipOFF system, *int* (trans), pANTO3::pANTO5 HygR^R^, Km^R^, Sm^R^	This study
TB213	TB167	P_*ptr*_-*sigA*, TetR- PipOFF system::pANTO5 HygR^R^, Sm^R^	This study
TB215	TB213	P_*ptr*_-*sigA*,::pANTO5,::pAGN33 *pip* merodiploid, Hyg^R^, Sm^R^, Km^R^	This study

To obtain conditional mutants merodiploid for *pip*, pAGN33 was electroporated into Ms165 and TB213 to obtain Ms199 and TB215, respectively (Table 2 and Table 3).

### Estimation of stability

*M*. *smegmatis* strains and *M*. *tuberculosis* strains were streaked on Middlebrook 7H10 plates with the appropriate antibiotics and two colonies for each strain were selected for further characterization. Each colony was used to inoculate 5 ml of Middlebrook 7H9; after 24 hrs (*M*. *smegmatis*) or 5 days (*M*. *tuberculosis*) of incubation at 37 °C, serial dilutions were plated with or without ATc (50 or 500 ng ml^−1^ for *M*. *smegmatis* or *M*. *tuberculosis*, respectively). The ratio of escape mutants was obtained dividing the number of colony forming units obtained in plates with ATc by the number of colony forming units obtained in plates without ATc.

### RNA extraction and quantitative RT-PCR

RNA extraction and quantitative reverse transcription real-time PCR (RT-PCR) were performed using Sybr Green Master Mix (Applied Biosystems) as previously described^4,15^ using *sigA* mRNA as internal invariant control^20^. RNA samples not subjected to reverse transcription were included exclude significant DNA contamination. Experiments, using independent biological samples were performed at twice.

## Results and Discussion

### Construction of a TetR/Pip OFF system with improved stability

**Figure 1:**
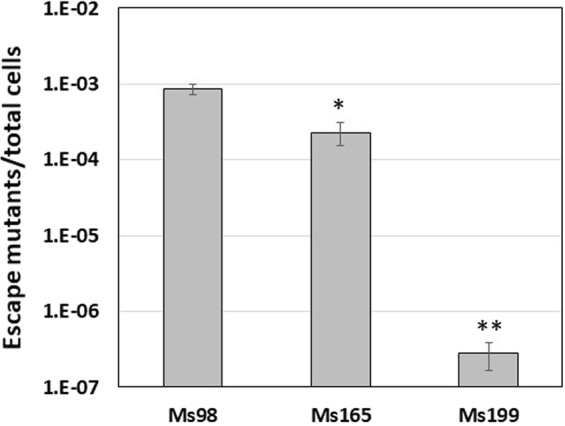
Ratio between escape mutants able to growth in ATc (50 ng/ml) and total cells population after 24 h of growth of different *M*. *smegmatis* mutants. The reported values derive from three independent experiments. *P < 0.05; **P < 0.01 versus Ms98 (Student’s *t*-test).

We previously reported the construction of a *M*. *smegmatis ftsZ* conditional mutant (MS98) in which *ftsZ* was placed under the transcriptional control of the repressible promoter P_*ptr*_. This strain has a plasmid integrated at its L5 *attB* site carrying the genes encoding for the TetR/Pip OFF system developed in our laboratory (pFRA42B) and conferring streptomycin (Sm) resistance^4^. This strain is able to grow in physiologic conditions, however addition of ATc to its culture medium leads to growth arrest and cell death due to *ftsZ* downregulation. It is known that L5-based integrative vectors can excise from the *attB* site and be lost from the recipient host. We previously showed that excision of pFRA42B from MS98 caused the selection of mutants escaping ATc-mediated *ftsZ* downregulation when this strain was grown in the absence of Sm^4^. The observed instability might represent a problem in long *in vivo* experiments, therefore we decided to improve the stability of the vector carrying the TetR/Pip OFF system. At this purpose we constructed two new plasmids: the first, named pANTO5, is an integrative plasmid bearing the TetR/Pip OFF system and the L5 *attP* site but not the L5 integrase gene and confers Sm resistance; the second, named pANTO3, is a replicative plasmid carrying the L5 integrase gene alone and conferring resistance to Km (Table 1). Both the replicative plasmid pANTO3 and the integrative plasmid pANTO5 were electroporated into *M*. *smegmatis* MS96 (progenitor of MS98) which carries the *ftsZ* gene under the transcriptional control of the repressible promoter P_*ptr*_ but lacks the integrative plasmid pFRA42B, and the resulting strain was named MS163 (Table 2). Integration was obtained thanks to the integrase provided in trans from pANTO3, since electroporation of pANTO5 alone into MS96 was not successful (data not shown). Subsequently, MS163 was grown in the absence of Km for about 4 generations and plated on Middlebrook 7H10 without Km. One hundred single colonies were then transferred to Middlebrook 7H10 plates containing Km to identify those strains that had lost pANTO3. A colony with the required phenotype was isolated and named MS165 (Table 2). The only difference between the *ftsZ* conditional mutant MS165 and the previously described MS98 is the absence of the gene encoding the L5 integrase. As a consequence, we expected that the excision of the integrative plasmid pANTO5 carrying the TetR/Pip OFF system in MS165 arose with lower frequency reducing therefore the occurrence of mutants escaping ATc repression. To confirm this hypothesis, both strains were grown in liquid cultures in the absence of Sm and plated on solid medium containing ATc. For MS98, the ratio of escape mutants/total cells was 8.5 × 10^−4^, whereas for MS165 this ratio was 2.3 × 10^−4^ (Fig. 1). One hundred escape mutants derived from each strain were further analysed for Sm resistance. In line with our hypothesis, the escape mutants coming from MS98 were all Sm sensitive, indicating the excision of the integrative plasmid (excision was PCR-verified in three mutants, results not shown), whereas those derived from MS165 were still resistant to this drug, indicating that ATc escape in this strain was not due to the loss of the integrative plasmid carrying *tetR* and *pip* genes but to some other mutations.

Escape mutants of MS165 could arise for the occurrence of mutations either in the operator of the P_*ptr*_ promoter upstream *ftsZ* that prevent the binding of the Pip repressor, or in the *pip* gene, preventing its expression or resulting in an inactive Pip protein. We therefore analysed the sequence of both the P_*ptr*_ promoter and *pip* in six randomly chosen MS165 escape mutants. None of the strains showed any mutations in the P_*ptr*_ promoter, however, in all of the 6 strains we found the insertion of a G in a stretch of 6 Gs at nucleotide 97 of *pip*, causing a frameshift mutation which consequently inactivated the gene (Fig. S2). In order to decrease the impact of these *pip* mutations on the stability of the system, we decided to create a *pip* merodiploid strain. To this purpose we cloned a copy of *pip* into pTTP1B, an integrative vector targeting the mycobacteriophage Tweety *attB* sites and conferring Km resistance^19^. The resulting plasmid was named pAGN33 (Table 1) and was introduced by electroporation in MS165 to obtain MS199 (Table 2). We then analysed the stability of this strain. As shown in Fig. 1, the ratio of escape mutants/total cells decreased impressively compared to the parental strain, dropping to 2.8 × 10^−7^. Ten colonies of MS199 escape mutants were further analysed for their phenotype and all of them were shown to have lost Km but not Sm resistance, indicating that in this case the occurrence of mutants was due to the excision of pAGN33 carrying the second copy of *pip* (excision of pAGN33 was confirmed by PCR, Figs S3 and S4).

### Evaluation of the stabilized TetR/Pip OFF system efficacy in repressing gene expression of target genes

**Figure 2:**
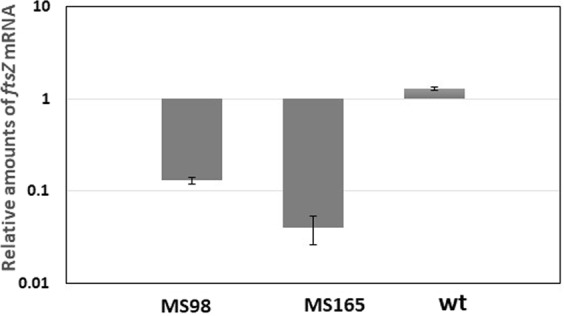
Changes in *ftsZ* mRNA levels upon exposure of exponentially growing cultures to 50 ng/ml ATc for 7 hours in the conditional *ftsZ* mutants MS98 and MS165 and in their parental wt strain mc^2^155. Values are expressed as the ratio between the number of cDNA copies detected in samples obtained from bacteria exposed to ATc and the number of cDNA copies detected in samples from parallel cultures not exposed to ATc. The values were normalized to the level of *mysA* cDNA, which represented the internal invariant control. The reported values derive from two indipendent experiments.

To evaluate the impact of the modifications made to stabilize the TetR/Pip OFF system on its efficacy in regulating the level of expression of the target genes, we evaluated by quantitative RT-PCR the level of expression of *ftsZ* in MS98, MS165 and MS199 using *mysA* (encoding the principal sigma factor of *M*. *smegmatis*) an internal invariant control. The results, clearly demonstrated that *ftsZ* was strongly repressed in all of the three strains. However, while repression in MS98 and MS165 was comparable (8 and 25 folds, respectively), and not significantly different (Fig. 2), the level of *ftsZ* mRNA in MS199 was below the level of detection of the amplification reaction (data not shown), indicating a dramatic increase of stringency due to the presence in this strain of two copies of the gene encoding the repressor Pip.

### Evaluation of the stabilized TetR/Pip OFF system in long term experiments

**Figure 3:**
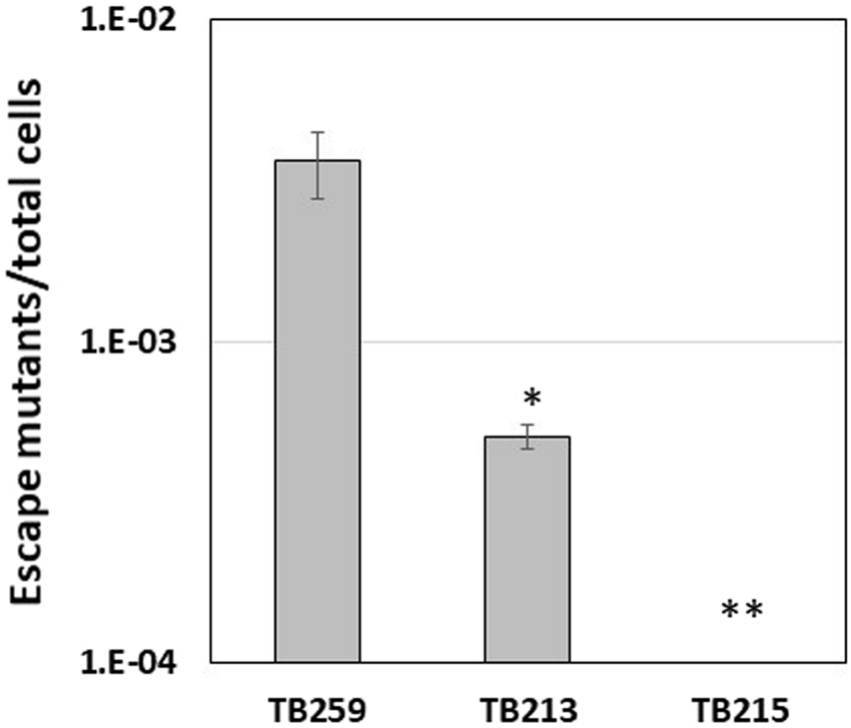
Ratio between escape mutants able to growth in ATc (500 ng/ml) and total cells population after 5 days of incubation of different *M*. *tuberculosis* mutants. The reported values derive from two independent experiments. *P < 0.05; **P < 0.01 versus TB259 (Student’s *t*-test).

Finally, we made an experiment to demonstrate that the use of MS199 allowed to perform long term experiments compared with MS98. At this purpose, MS98 and MS199 were grown in liquid culture in the presence of ATc. To correct for ATc decay in the medium fresh ATc was added every 24 h^6^. After 24 h of incubation the culture of both strains showed a marked decrease of the optical density due to clumping of the bacteria as previously shown for MS98^4^. After additional 24 hours of incubation we could notice a marked increase of optical density due to the selection of escape mutants only in the MS98 culture, while growth in the MS199 culture did not resume at least until 72 hours of incubation (Fig. 3).

### Evaluation of the stabilized TetR/Pip OFF system in *M*. *tuberculosis*

**Figure 4:**
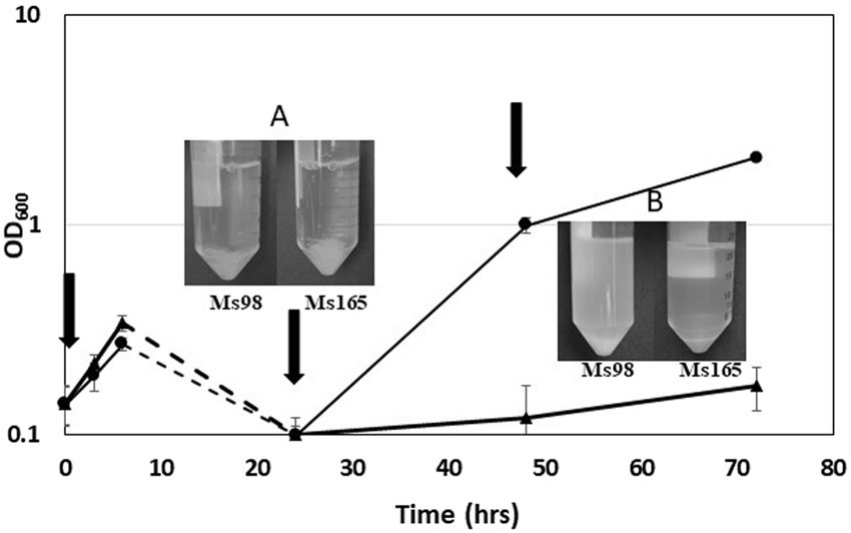
Growth curve of Ms98 and Ms165 ATc 50 ng/ml. ATc was added at T0, T24 and T48 to provide continue *ftsZ* repression (arrows). (**A**) Effects of *ftsZ* gene repression after 24 hours of incubation in the presence of ATc: clumping of both mutants is visible on the bottom of the tubes; (**B**) Effects of *ftsZ* gene repression after 48 hours of incubation in the presence of ATc: growth resumption in MS98 culture, but not in MS165 culture is visible. Data represent the average of two independent experiments. Circles: MS98; triangles: MS165.

The stability of the system was then analysed in *M*. *tuberculosis*. To this purpose we used a conditional mutant obtained in our lab by replacing the *sigA* promoter with the P_*ptr*_ promoter (manuscript in preparation) (TB259) (Table 3). SigA is the principal mycobacterial sigma factor, it was previously shown to be essential in *M*. *smegmatis*^21,22^, and was hypothesized to be essential also in *M*. *tuberculosis*. In line with this hypothesis, when TB259 was exposed to ATc, *sigA* expression was repressed and the bacteria stopped growing (manuscript in preparation). Following the same experimental design used for *M*. *smegmatis*, we first constructed a strain with the TetR/Pip-OFF system integrated into the chromosome of *M*. *tuberculosis* and devoid of integrase to stabilize its insertion (TB213) (Table 3) as described in Materials and Methods. Subsequently, TB213 was transformed with pAGN33 carrying an additional copy of *pip* to obtain TB215 (Table 3). All the three strains were then grown in the absence of selection for 5 days and plated with or without ATc. As expected, the largest fraction of escape mutants/total cells able to grow in the presence of ATc was selected from the original mutant TB259 (3.63 × 10^−3^). As for TB213 devoid of the integrase, the stability of the integrative plasmid carrying the TetR/Pip-OFF system was higher, with a ratio of escape mutants/total cells of 5.04 × 10^−4^. The strain showing the highest stability was TB215, the derivative merodiploid for *pip* for which we could not observe any escape mutant (Fig. 4).

## Conclusions

In this paper we showed that the instability of the TetR/Pip-OFF system was due either to the loss of the integrative plasmid carrying the *tetR* and *pip* genes or to the selection of a frameshift mutation on the *pip* gene. The stabilization of the integrative plasmid carrying the *tetR* and *pip* genes through the deletion of the integrase gene and the introduction of a second copy of the *pip* gene on the chromosome of the mutant, drastically decreased the previously observed instability and increased the stringency of the system making it more suitable for long term experiments.

## Supplementary information

Supplementary Information
