# Supplementary material for: Improving the stability of the TetR/Pip-OFF mycobacterial repressible promoter system
Source: Sci Rep. 2019 Apr 8;9:5783. doi: 10.1038/s41598-019-42319-2 (PMC6453970; doi:10.1038/s41598-019-42319-2)
Supplement: Supplementary file 1 — Supplementary Information [file 41598_2019_42319_MOESM1_ESM.pdf]

Improving the stability of the TetR/Pip-OFF mycobacterial repressible promoter system

Francesca Boldrin[1], Saber Anoosheh[1§], Agnese Serafini[1Φ], Laura Cioetto Mazzabò[1], Giorgio Palù[1], Roberta Provvedi[2], Riccardo Manganelli[1*]

[1]Department of Molecular Medicine; [2] Department of Biology, University of Padova, Padova, Italy.

[§]**Present address:** Institute of Infectious Disease and Molecular Medicine, University of Cape Town UCT, Cape Town, South Africa.

[Φ]**Present address:** Mycobacterial Metabolism and Antibiotic Research Laboratory, The Francis Crick Institute, London, UK.

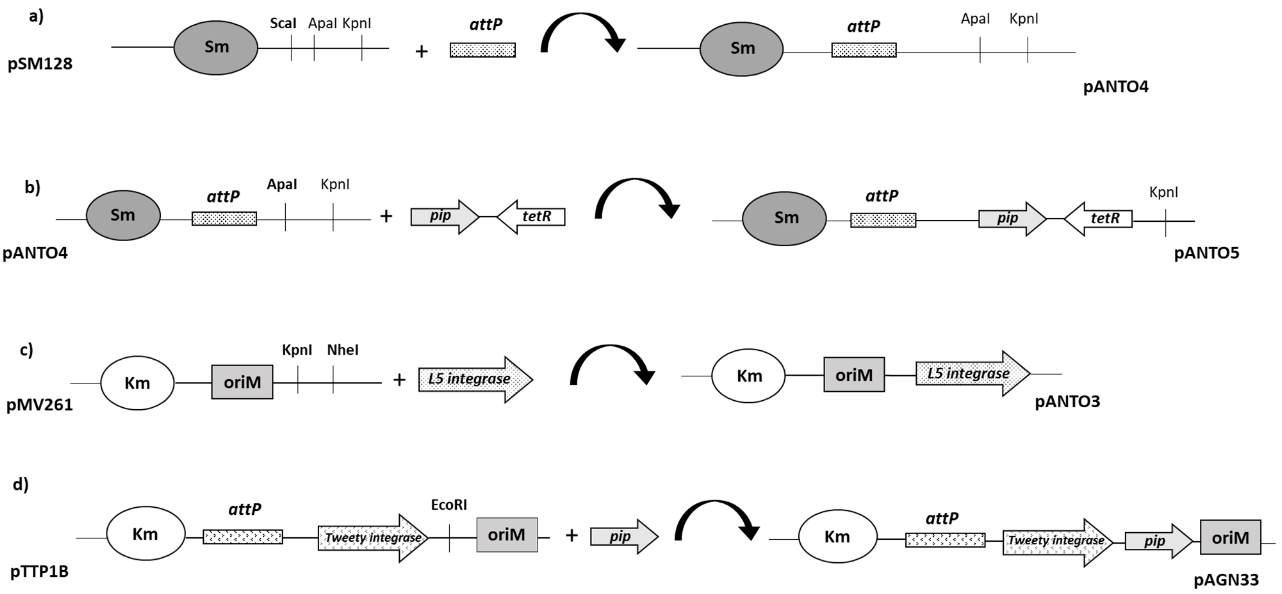

Figure S1. Schematic representation of plasmids construction. a) pANTO4 was obtained cloning the bacteriophage L5 *attP* site into the ScaI site of pSM128; b) pANTO5 was obtained cloning *tetR-pip* genes in the ApaI site of pANTO4; c) pANTO3 was obtained by cloning the bacteriophage L5 integrase gene between the KpnI and NheI sites of pMV261; d) pAGN33 was obtained cloning the *pip* gene into the EcoRI site of pTTP1B. Explanations in the text.

```
1) GATCCAGCGGCCCCGGGGCGATCGGGTCCTAGCAGACGCCTCCCTATCAGTGATAGATCA
2) GATCCAGCGGCCCCGGGGCGATCGGGTCCTAGCAGACGCCTCCCTATCAGTGATAGATCA
   ************************************************************

1) ACTGATCCCTATCAGTGATAGACATATTGTCTAGTGTGGCGGCCGCGAGTCGAGGAGAGG
2) ACTGATCCCTATCAGTGATAGACATATTGTCTAGTGTGGCGGCCGCGAGTCGAGGAGAGG
   ************************************************************

1) TGCGCATGGCGAAGGCAGGGCGGGAGGGGCCGCGGGACAGCGTGTGGCTGTCGGGGGAGG
2) TGCGCATGGCGAAGGCAGGGCGGGAGGGGCCGCGGGACAGCGTGTGGCTGTCGGGGGAGG
   ************************************************************

1) GGCGGCGCGGCGGTCGCCGTGGGGGGGCAGCCGTCCGGGCTCGACCGGGACCGGATCACC
2) GGCGGCGCGGCGGTCGCCGTGGGGGG-CAGCCGTCCGGGCTCGACCGGGACCGGATCACC
   ************************** *********************************

1) GGGGTCACCGTCCGGCTGCTGGACACGGAGGGCCTGACGGGGTTCTCGATGCGCCGCCTG
2) GGGGTCACCGTCCGGCTGCTGGACACGGAGGGCCTGACGGGGTTCTCGATGCGCCGCCTG
   ************************************************************

1) GCCGCCGAGCTGAACGTCACCGCGATGTCCGTGTACTGGTACGTCGACACCAAGGACCAG
2) GCCGCCGAGCTGAACGTCACCGCGATGTCCGTGTACTGGTACGTCGACACCAAGGACCAG
   ************************************************************

1) TTGCTCGAGCTCGCCCTGGACGCCGTCTTCGGCGAGCTGCGCCACCCGGACCCGGACGCC
2) TTGCTCGAGCTCGCCCTGGACGCCGTCTTCGGCGAGCTGCGCCACCCGGACCCGGACGCC
   ************************************************************

1) GGGCTCGACTGGCGCGAGGAACTGCGGGCCCTGGCCCGGGAGAACCGGGCGCTGCTGGTG
2) GGGCTCGACTGGCGCGAGGAACTGCGGGCCCTGGCCCGGGAGAACCGGGCGCTGCTGGTG
   ************************************************************

1) CGCCACCCCTGGTCGTCCCGGCTGGTCGGCACCTACCTCAACATCGGCCCGCACTCGCTG
2) CGCCACCCCTGGTCGTCCCGGCTGGTCGGCACCTACCTCAACATCGGCCCGCACTCGCTG
   ************************************************************

1) GCCTTCTCCCGCGCGGTGCAGAACGTCGTGCGCCGCAGCGGGCTGCCCGCGCACCGCCTG
2) GCCTTCTCCCGCGCGGTGCAGAACGTCGTGCGCCGCAGCGGGCTGCCCGCGCACCGCCTG
   ************************************************************

1) ACCGGCGCCATCTCGGCCGTCTTCCAGTTCGTCTACGGCTACGGCACCATCGAGGGCCGC
2) ACCGGCGCCATCTCGGCCGTCTTCCAGTTCGTCTACGGCTACGGCACCATCGAGGGCCGC
   ************************************************************

1) TTCCTCGCCCGGGTGGCGGACACCGGGCTGAGTCCGGAGGAGTACTTCCAGGACTCGATG
2) TTCCTCGCCCGGGTGGCGGACACCGGGCTGAGTCCGGAGGAGTACTTCCAGGACTCGATG
   ************************************************************

1) ACCGCGGTGACCGAGGTGCCGGACACCGCGGGCGTCATCGAGGACGCGCAGGACATCATG
2) ACCGCGGTGACCGAGGTGCCGGACACCGCGGGCGTCATCGAGGACGCGCAGGACATCATG
   ************************************************************

1) GCGGCCCGGGGCGGCGACACCGTGGCGGAGATGCTGGACCGGGACTTCGAGTTCGCCCTC
2) GCGGCCCGGGGCGGCGACACCGTGGCGGAGATGCTGGACCGGGACTTCGAGTTCGCCCTC
   ************************************************************

1) GACCTGCTCGTCGCGGGCATCGACGCGATGGTCGAACAGGCCTGA
2) GACCTGCTCGTCGCGGGCATCGACGCGATGGTCGAACAGGCCTGA
   *********************************************
```

Figure S2. CLustalW allignment between the wt *pip* sequence cloned into pFRA61 (1) and the corresponding sequence in one of the 6 selected Ms165 mutant colonies grown in the presence of ATc (50 ng/ml). -35 and – 10 sequences are underlined; the *pip* start codon AGT is shown in bold; the insertion of a G in a stretch of 6 Gs at nucleotide 97 of *pip* is indicated by an arrow.

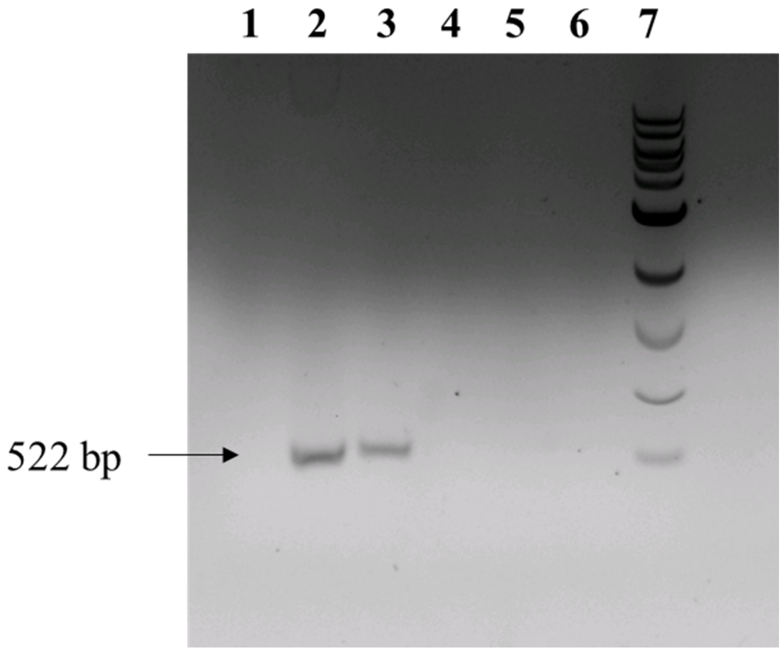

Figure S3. Agarose gel electrophoresis to confirm pAGN33 loss in three MS199 ATc mutants. Samples were amplified using pAGN33 specific primers RP1982 (5'GATGCGCCAGAGTTGTTTCT 3') and RP1983 (5' TCCGACTCGTCCAACATCAA). 1) NO DNA control; 2) pAGN33 DNA; 3) genomic DNA of Ms199; 4-6: genomic DNA of three Ms199 Km sensitive mutants; 7) 1 Kb ladder. A PCR product of the expected size is present only in lines 2 and 3, representing pAGN33 and MS199 genome (in which pAGN33 is integrated), respectively. However, no PCR product is visible in lanes 4, 5, and 6 representing MS199 escape mutants showing the loss of pAGN33 from their chromosomes. See Figure S4 for an uncropped version of this figure.

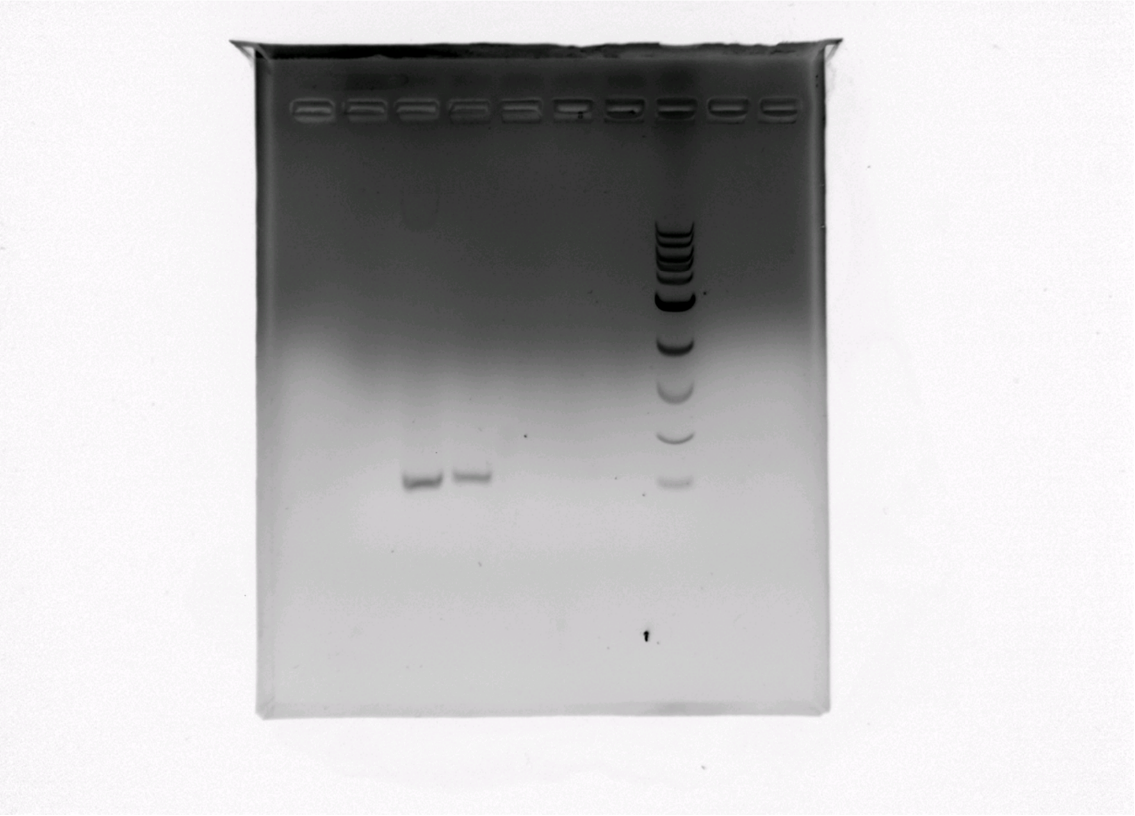

Figure S4. Uncropped version of Figure S3.
